# Elucidation of the Crystal Growth Characteristics of SnO_2_ Nanoaggregates Formed by Sequential Low-Temperature Sol-Gel Reaction and Freeze Drying

**DOI:** 10.3390/nano11071738

**Published:** 2021-07-01

**Authors:** Saeid Vafaei, Alexander Wolosz, Catlin Ethridge, Udo Schnupf, Nagisa Hattori, Takashi Sugiura, Kazuhiro Manseki

**Affiliations:** 1 Mechanical Engineering Department, Bradley University, 1501 West Bradley Avenue, Peoria, IL 61625, USA; awolosz@mail.bradley.edu (A.W.); cethridge@mail.bradley.edu (C.E.); 2 Mund-Lagowski Department of Chemistry and Biochemistry, Bradley University, 1501 West Bradley Avenue, Peoria, IL 61625, USA; uschnupf@fsmail.bradley.edu; 3 Graduate School of Natural Science and Technology, Gifu University, Gifu 501-1193, Japan; a4524054@edu.gifu-u.ac.jp (N.H.); tsugiura@gifu-u.ac.jp (T.S.)

**Keywords:** nanoparticles, SnO_2_, crystallization, freeze drying, low-temperature synthesis, oxygen vacancies

## Abstract

SnO_2_ nanoparticles are regarded as attractive, functional materials because of their versatile applications. SnO_2_ nanoaggregates with single-nanometer-scale lumpy surfaces provide opportunities to enhance hetero-material interfacial areas, leading to the performance improvement of materials and devices. For the first time, we demonstrate that SnO_2_ nanoaggregates with oxygen vacancies can be produced by a simple, low-temperature sol-gel approach combined with freeze-drying. We characterize the initiation of the low-temperature crystal growth of the obtained SnO_2_ nanoaggregates using high-resolution transmission electron microscopy (HRTEM). The results indicate that Sn (II) hydroxide precursors are converted into submicrometer-scale nanoaggregates consisting of uniform SnO_2_ spherical nanocrystals (2~5 nm in size). As the sol-gel reaction time increases, further crystallization is observed through the neighboring particles in a confined part of the aggregates, while the specific surface areas of the SnO_2_ samples increase concomitantly. In addition, X-ray photoelectron spectroscopy (XPS) measurements suggest that Sn (II) ions exist in the SnO_2_ samples when the reactions are stopped after a short time or when a relatively high concentration of Sn (II) is involved in the corresponding sol-gel reactions. Understanding this low-temperature growth of 3D SnO_2_ will provide new avenues for developing and producing high-performance, photofunctional nanomaterials via a cost-effective and scalable method.

## 1. Introduction

Over the last two decades, the synthesis of self-assembled tin oxide (SnO_2_) semiconductor nanoparticles has been a fascinating research area because of their potential applications in gas sensing, lithium-ion batteries, solar cells, and catalysts for various organic reactions [1]. These versatile applications associated with their photophysical, chemical, and electronic properties can be realized by tuning certain factors, such as the size, shape, crystallinity, and electronic states of the SnO_2_ nanoparticles [2]. In particular, self-assembled 3D SnO_2_ nanoaggregates with high surface areas [3,4] have received considerable attention. A bottom-up, wet-chemical synthesis is one of the major techniques that enables the creation of nanoparticles with various dimensions, ranging from spherical morphologies [5] to anisotropic 1D structures [6], 2D sheets [7], and self-assembled 3D forms [1] of low-dimensional motifs. Thus far, such SnO_2_ production has relied heavily on hydrothermal synthesis, which requires a high temperature and pressure. As an example, to form the target hierarchical nanostructures, a conventional hydrothermal approach has been reported to produce a 3D flower-like SnO_2_ nano/microstructure material [8]. From the SnCl_4_ precursor solution with a poly (acrylic acid) additive, flower-like particles with a dimension of 100 nm consisting of needle-like single-crystal units (ca. 50 nm in size) were obtained under acidic conditions at 150 °C. Several other syntheses of SnO_2_ particles with flower-like morphology have also been reported to date, using various Sn (II) precursors under hydrothermal conditions [9,10].

A low-temperature solution process employing a sol-gel method is considered an alternative method for creating oxide nanoparticles with high surface areas. This is because, in this method, nucleation and crystal growth at a single nanometer scale can be controlled by the selection of base solvents, metal-ion sources, and organic additives, compared to the cases in the well-known hydrothermal or solvothermal methods. Among wide-bandgap semiconductor metal oxides, several controlled low-temperature hydrolyses and polycondensations in TiO_2_ syntheses have been shown to produce TiO_2_ nanoparticles of different sizes, crystal phases, and morphologies, with dimensions of less than 10 nm [11,12]. We recently demonstrated that organic-inorganic Ti (IV)-gels prepared at temperatures as low as 40 °C could be used as a precursor to obtain anatase TiO_2_ nanoaggregates consisting of particles with a grain size of 5 nm [13]. Freeze drying of the gel precursor accelerates the polycondensation in the Ti-O framework to efficiently produce nanocrystals even at low temperatures.

In contrast to the several low-temperature TiO_2_ syntheses reported in the literature, the growth mechanism of SnO_2_ nanoparticles and their 3D structure formation at low temperatures have not been adequately understood to date. SnO_2_ films processed at relatively low temperatures are regarded as attractive materials with the progress of new-generation solar cells, specifically halide perovskite solar cells [14,15,16,17,18]. Therefore, fundamental research regarding SnO_2_ crystallization is essential for developing electron-transport materials that boost device performance.

In this study, simple template-free reaction conditions employing SnCl_2_ and Na_2_CO_3_ in water were applied to investigate a low-temperature SnO_2_ crystal growth, along with freeze-drying. The formation of 3D SnO_2_ nanoaggregates with uniform, single nanometer-scale grains, and their crystallization were clarified, mainly using reaction time-dependent, high-resolution HRTEM analysis. For the first time, we showed the low-temperature crystal growth characteristics of SnO_2_ nanoaggregates involving the formation of oxygen vacancies. The detailed crystallization of the pre-organized Sn-based gel precursors was assessed to form 3D SnO_2_ nanoaggregates with controllable microstructure surfaces and electronic properties.

## 2. Materials and Methods

### 2.1. Synthesis of SnO_2_ Nanoaggregates

SnCl_2_·2H_2_O and Na_2_CO_3_ were purchased from Sigma-Aldrich, St. Luis, Missouri, USA and used as received. Three different synthesis methods were applied to synthesize the semiconductor SnO_2_ nanoparticles. The mass ratio of ultrapure water (UPW), SnCl_2_, and Na_2_CO_3_ were varied, as shown in Methods 1–3 below.

Method 1: The precursor solution was prepared by mixing SnCl_2_ (2 g) dissolved in UPW (400 mL). Na_2_CO_3_ (400 mg) was dissolved separately in UPW (500 mL) and completely mixed with the SnCl_2_ solution. The solution was placed in a water bath and maintained at 40 °C for a given time, denoted as Day X (X: 1–7 or 1–8) hereinafter. The SnO_2_ solution was sampled by transferring 150 mL to a centrifugation container. Thereafter, the container was centrifuged at 4000 rpm for 10 min. The reaction temperature was maintained at 40 °C. After centrifugation, the liquid was discarded. The solid gel was carefully mixed in 5–10 mL of water and sonicated for uniform dispersion. Subsequently, it was added to a vial and capped. The vial was placed in a container filled with liquid nitrogen for 5–10 min to freeze the gel solution. Afterward, the vial was placed into a jar and attached to a vacuum freeze-drying apparatus (BenchTop Pro with Omnitronics, VirTis SP Scientific, USA) at ~200 mTorr. After 24–48 h of freeze-drying to remove the moisture, SnO_2_ nanoparticles were produced in a powdered form.

Method 2: The precursor solution was prepared using a mixture of SnCl_2_ (5 g) dissolved in UPW (500 mL). Na_2_CO_3_ (500 mg) was dissolved separately in UPW (500 mL) and completely mixed with the SnCl_2_ solution. Thereafter, the solution was placed in a water bath and maintained at 40 °C for a given period. The SnO_2_ nanoparticles were isolated using a similar procedure to that in Method 1.

Method 3: The precursor solution was prepared by dissolving SnCl_2_ (20 g) in UPW (500 mL), in contrast to using 5 g of SnCl_2_, as in Method 2. The SnO_2_ nanoparticles were isolated using a similar procedure to that in Method 1.

### 2.2. Structure Characterization of SnO_2_ Nanoaggregates

SnO_2_ powder samples were characterized using X-ray diffraction (XRD; Rigaku RINT Ultima/PC with monochromated Cu–Kα radiation, Tokyo, Japan). The crystallite size of the SnO_2_ aggregates was estimated using the Scherrer equation (D = Kλ/βcosθ) based on the XRD data, where D, K, λ, and θ indicate the crystallite size, Scherrer constant (0.90), X-ray wavelength (1.54 Å), and Bragg angle, respectively. The SnO_2_ sample surface was analyzed by XPS (XPS; ULVAC, Quantera SXM, Kanagawa, Japan). Nanostructure analysis was carried out by TEM (JEM-2100, Tokyo, Japan). The Brunauer–Emmett–Teller (BET) surface area was evaluated by N_2_ physisorption measurement at 77 K, using the Micromeritics TriStar II 3020 (Kyoto, Japan). Photoabsorption spectra were measured using a Hitachi U-4000 spectrophotometer. Ultraviolet–visible (UV–Vis) spectra were obtained from the diffuse reflectance of the dry powder samples.

## 3. Results

### 3.1. XRD Patterns and XPS Spectra of the Obtained Powder Samples

**Figure 1:**
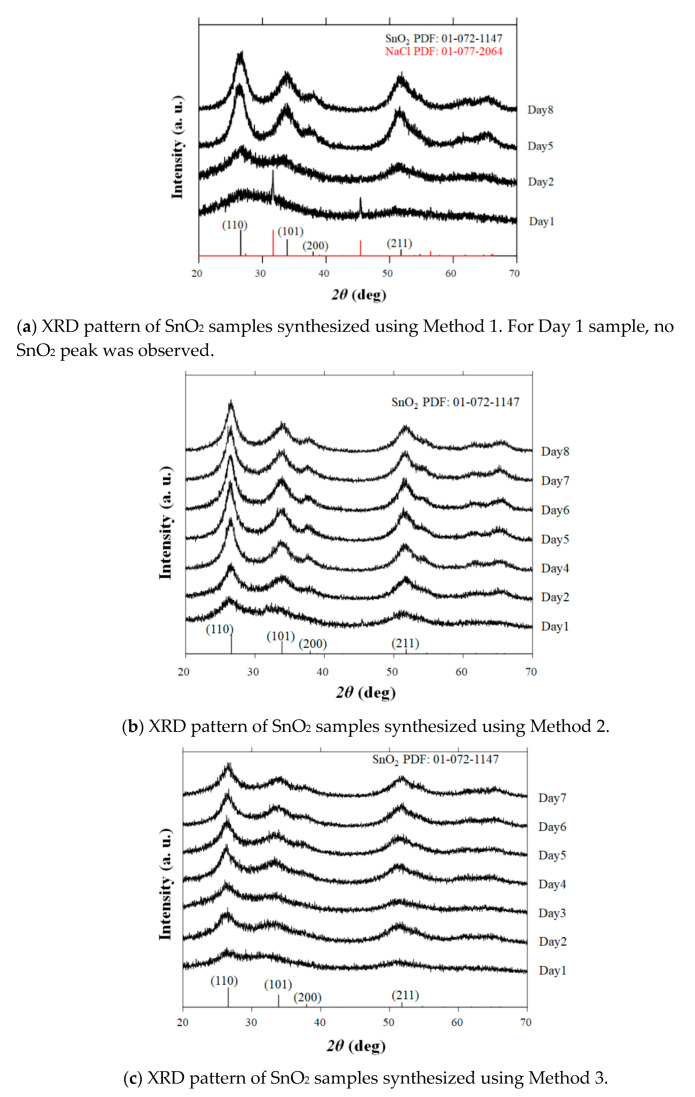
Powder X-ray diffraction (XRD) patterns of Method 1–3 samples synthesized by varying the reaction times. The database pattern is presented at the bottom of each figure.

We previously reported that anatase TiO_2_ nanoaggregates can be produced by freeze drying using Ti-gel precursors [13]. This result stimulated our interest in the growth control of SnO_2_ nanoaggregates at low temperatures. The XRD patterns of the powder samples obtained using three sets of reactions (Methods 1–3) are shown in Figure 1a–c. All the observed peaks are in agreement with those of tetragonal rutile SnO_2_ (JCPDS 01-072-1147), except for the Method 1–Day 1 sample. In the early stage of the sol–gel process in Method 1, only the impurity phase of NaCl is detected, and no SnO_2_ peak is observed. All the peaks corresponding to SnO_2_ are identified for the rest of the samples obtained via Method 1. It is observed that the intensity of the SnO_2_ peaks increases with the reaction times for all methods, indicating that long reaction times lead to the enhancement of the SnO_2_ crystal growth. 

**Figure 2:**
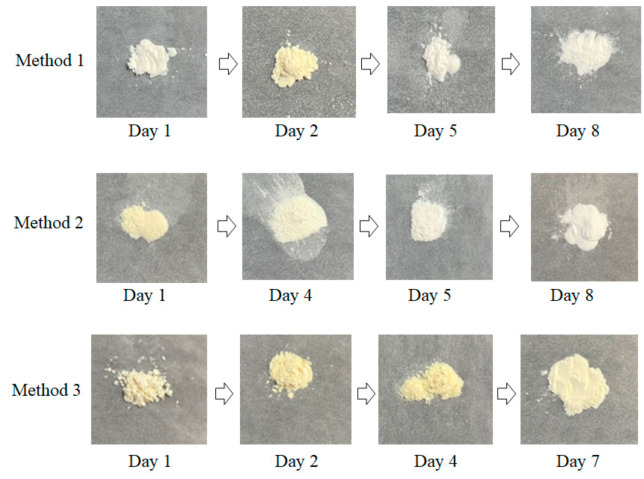
Photographs of the obtained representative powder samples synthesized by a sequential low-temperature sol–gel and freeze-drying approach.

**Figure 3:**
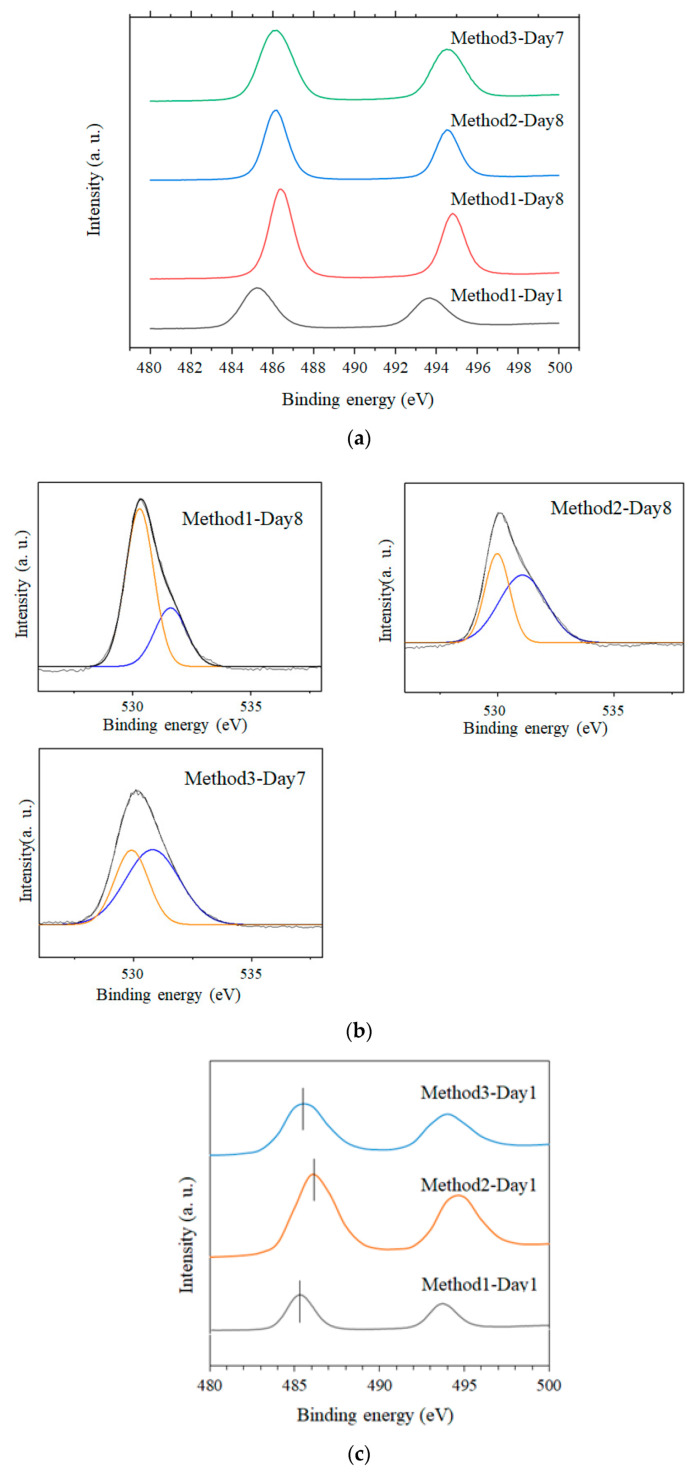
(**a**) X-ray photoelectron spectroscopy (XPS) spectra of Method 1–Day 1, Method 1–Day 8, Method 2–Day 8, and Method 3–Day 7 samples corresponding to Sn 3d5/2; (**b**) XPS spectra of the same samples (except Method 1–Day 1) showing O 1s peaks; (**c**) XPS spectra of Method 3–Day 1, Method 2–Day 1, and Method 1–Day 1 samples.

The samples obtained via Methods 1 and 2 exhibited a pale-yellow color when the solgel reactions were stopped in a short time (except for the Method 1–Day 1 samples, which exhibited a white color), and became white when the reactions were extended. Conversely, all the samples for Method 3 had a pale-yellow color (Figure 2). To understand the color-change tendency, XPS measurements of Method 1–Day 1, Method 1–Day 8, Method 2–Day 8, and Method 3–Day 7 samples were carried out as illustrated in Figure 3a,b. The binding energies corresponding to the peaks of Sn 3d5/2 for the Day 7 and 8 samples were 486.2, 486.4, and 486.2 eV for Method 3–Day 7, Method 1–Day 8, and Method 2–Day 8, respectively, which are almost consistent with the reported values for Sn(IV) bound to an oxygen atom in SnO_2_ [19,20,21]. Notably, all three pale-yellow samples showed a marked peak shift compared to that of the Method 1–Day 1 sample. For the Method 1–Day 1 sample, the Sn3d XPS peaks were observed at a lower binding energy (485.2 eV) than those for the other samples, suggesting that the Sn(II) in this sample was not oxidized to Sn(IV). Similarly, a peak shift was observed for the pale-yellow sample (Method 2–Day 1) when compared to that of Method 1–Day 1, as shown in Figure 3c.

The high-resolution O 1s spectra from the same sample could be deconvoluted into two peaks at approximately 530–532 eV, as indicated by the orange and blue lines. It has been reported that the low energy peak is ascribed to the oxygen bound to Sn atoms, whereas the high energy peak corresponds to the formation of oxygen vacancies [22,23]. Considering that SnO_2_ peaks were only detected in the XRD patterns, the large amount of Sn(II) source for Method 3 probably enhanced the formation of oxygen-vacancy-induced SnO_2_, even for long reactions.

### 3.2. Proposed Growth Mechanism of SnO_2_ Based on TEM Analysis and Its Optical Property

**Figure 4:**
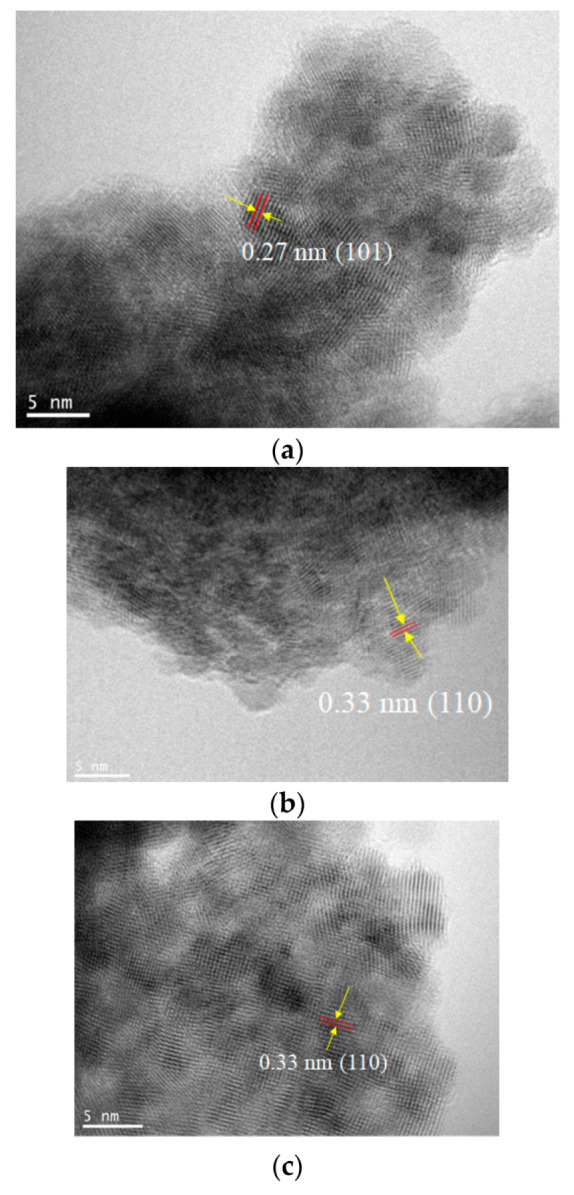

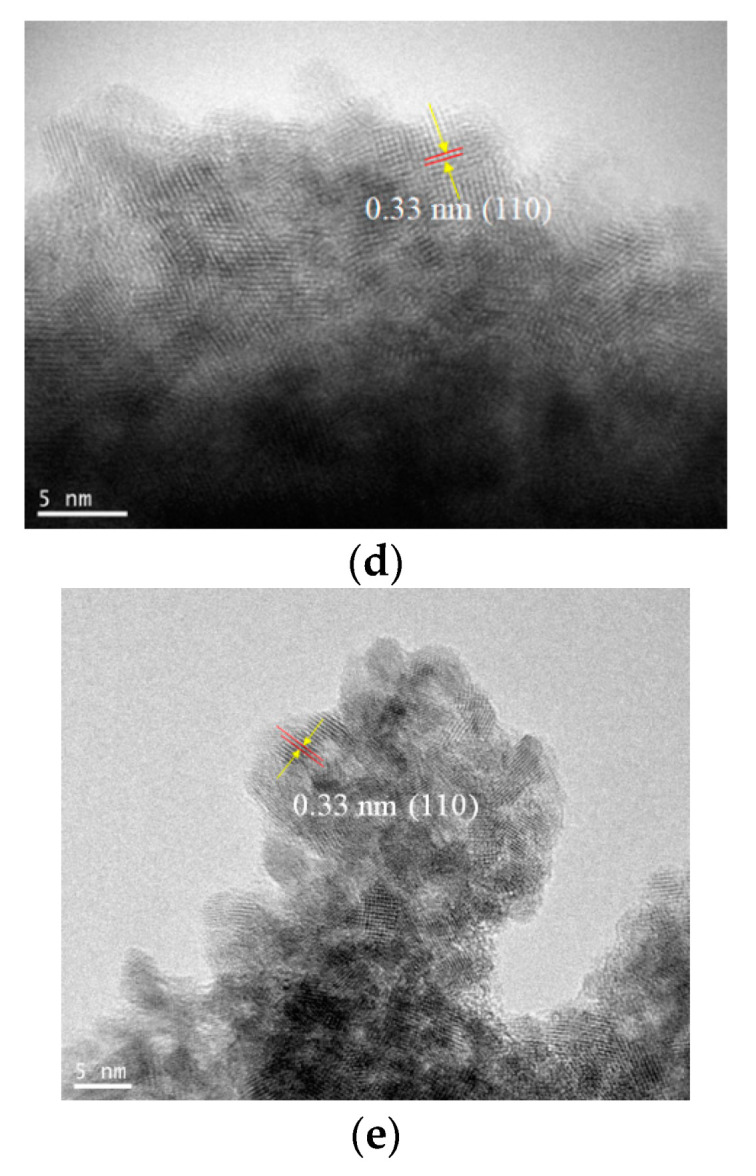
Transmission electron microscopy (TEM) images of (**a**) Method 1–Day 5, (**b**) Method 2–Day 1, (**c**) Method 2–Day 5, (**d**) Method 3–Day 1, and (**e**) Method 3–Day 5 samples.

**Figure 5:**
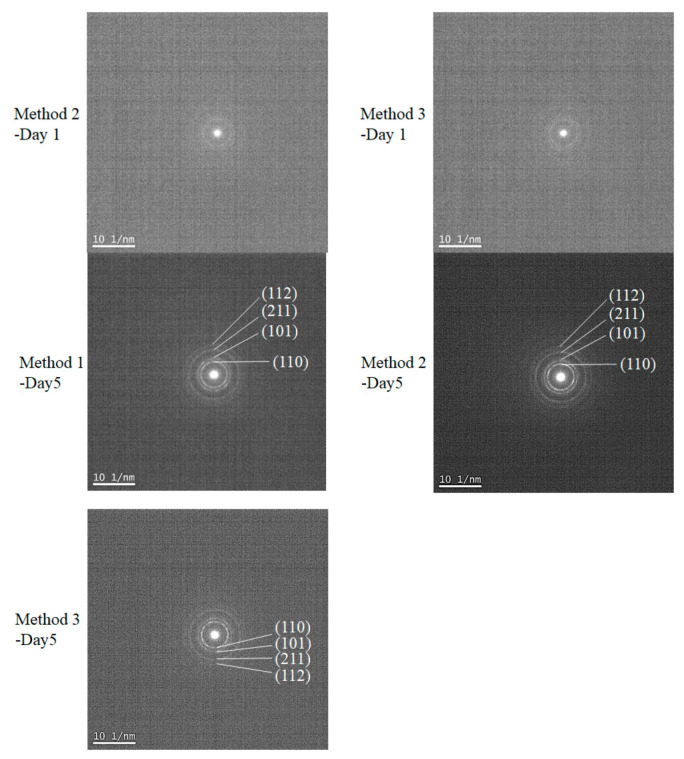
Selected area diffraction (SAD) pattern of Method 1–Day 5, Method 2–Day 1, Method 2–Day 5, Method 3–Day 1, and Method 3–Day 5 samples. These were measured using the same samples as those in Figure 4, which shows the TEM images.

To resolve the SnO_2_ nanostructures and elucidate their growth mechanism, HRTEM measurements were performed, as shown in Figure 4. The crystallization was not significant for all early-stage samples, such as the Method 1–Day 1 sample, as shown in the selected area diffraction (SAD) pattern (not shown). As the reaction times increase, lattice fringes with d-spacings of 0.33 nm and 0.27 nm corresponding to the (110) and (101) planes, respectively, are clearly observed for all methods. The measurements indicated that the aggregated particles consisting of tiny nanocrystals were formed from precursor Sn(II)-based gels, where the average sizes of the primary nanocrystals were in the range of 2~5 nm (Table 1), which are consistent with the estimated crystallite sizes in Table 2. Notably, the diffraction spots in SAD patterns become more noticeable as the reaction proceeds, although the corresponding measured area showed numerous tiny nanoparticles, as shown in Figure 5. This further indicated the improved crystallinity of the long-reaction-time samples.

**Figure 6:**
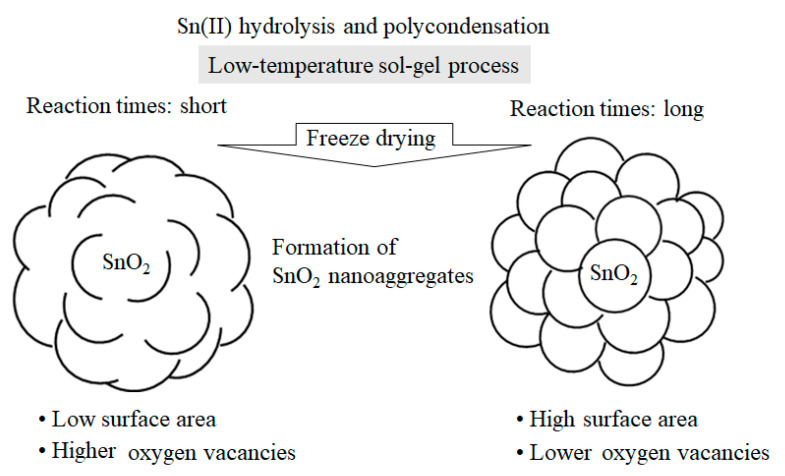
Plausible crystal growth mechanism of SnO_2_ nanoaggregates depending on reaction times of the low-temperature sol–gel process.

In addition, it was observed that the size of the SnO_2_ nanocrystals decreased slightly as the SnCl_2_ amount increased (Table 2). In our low-temperature process, the amount of SnCl_2_ was another key factor in controlling the size of the primary SnO_2_ nanoparticles, along with their crystallinity and generation rate of oxygen vacancies. As for the growth mechanism of the SnO_2_ particles, it is most probable that Sn(II) hydroxide precursors are formed by the hydrolysis of SnCl_2_ and subsequently converted into submicrometer-scale nanoaggregates comprising uniform SnO_2_ spherical nanocrystals. Figure 6 shows a conceptual drawing of the crystal growth characteristics of the SnO_2_ nanoaggregates in our low-temperature sol–gel reaction of Sn(II) and the subsequent freeze-drying process. The longer the sol–gel reaction time, the more enhanced the crystallization through its neighboring particles in a confined part of the nanoaggregates, while the specific surface areas of the SnO_2_ samples increase. In other words, long reactions produce SnO_2_ nanoaggregates with single-nanometer-scale lumpy surfaces, providing increased specific surface areas.

For Methods 2 and 3, the specific surface areas for Days 2 and 5 were almost comparable, suggesting that both SnO_2_ nanoaggregates for Day 2 already had lumpy surfaces, as illustrated on the right in Figure 6. Therefore, it is most likely that the SnO_2_ nanoaggregates after 3–4 days had surfaces similar to the image on the right. For Method 1, it would also be reasonable that, with increasing the reaction times, the SnO_2_ surface morphology changes from left to right, as shown in Figure 6. However, the surface morphology after 3–4 days cannot be explained solely by the change of the specific surface areas between Day 2 and Day 5. More detailed nanostructure analyses of the assembled SnO_2_ particles, i.e., 3D grain growth, will benefit from theoretical/computational modeling, in combination with the HRTEM data of SnO_2_ particles [24].

**Figure 7:**
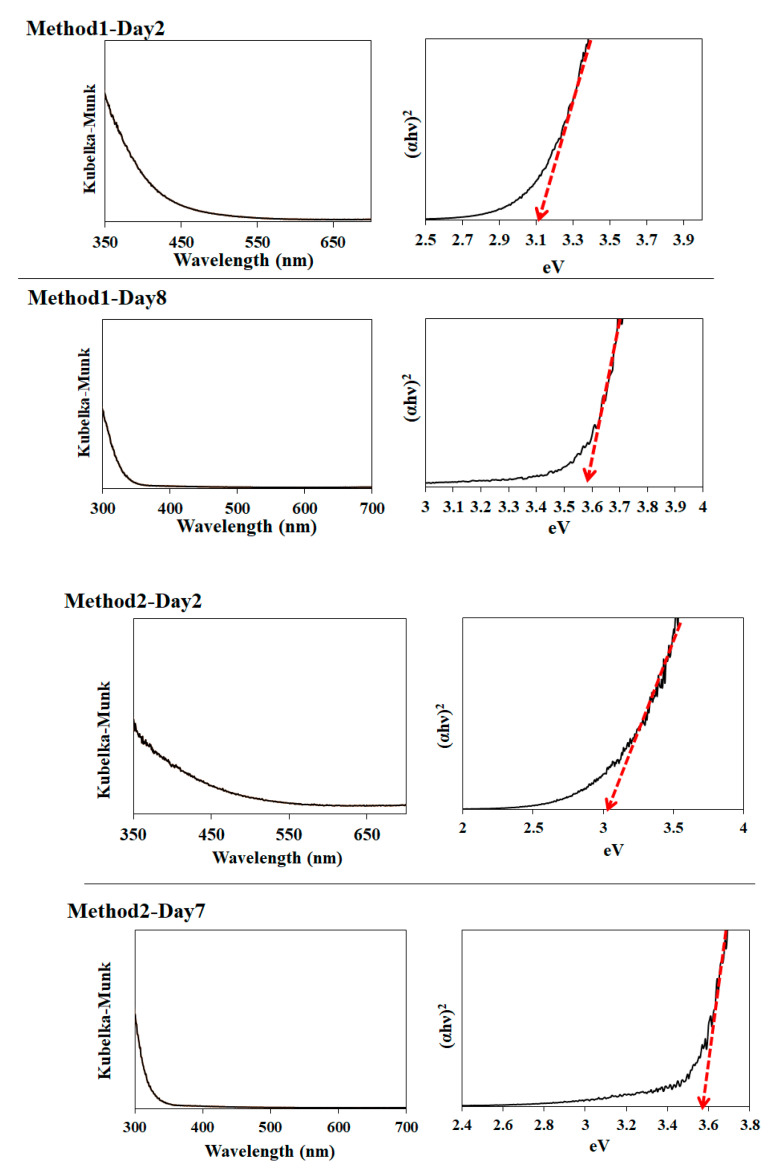

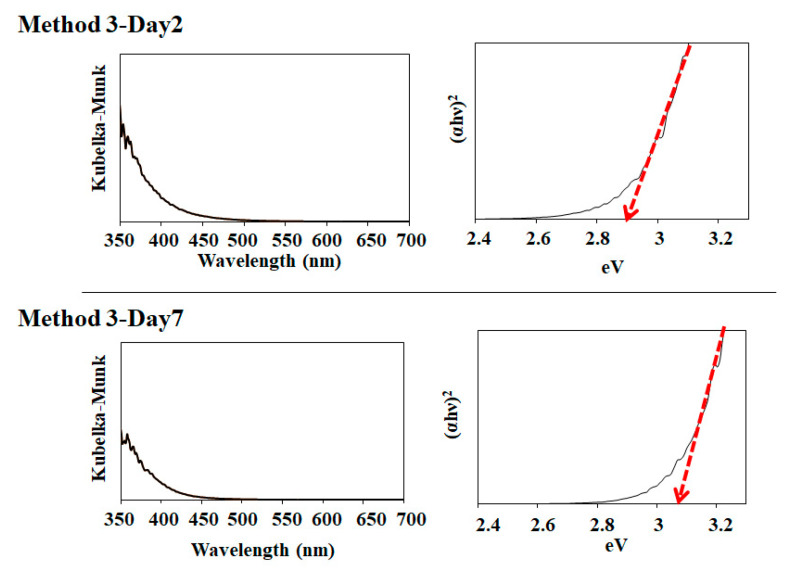
Photoabsorption spectra of Method 1–Day 2, Method 1–Day 8, Method 2–Day 2, Method 2–Day 7, Method 3–Day 2, Method 3–Day 7 samples and their corresponding bandgap estimations.

Additionally, we measured the optical absorption of several SnO_2_ samples of Methods 1–3 using diffuse reflectance spectroscopy, as shown in Figure 7. Notably, the samples of Method 1–Day 2, Method 2–Day 2, Method 3–Day 2, and Method 3–Day 7 showed an absorption tail in the visible wavelength. Their observed bandgaps were estimated to be 3.1, 3.0, 2.9, and 3.1 eV for Method 1–Day 2, Method 2–Day 2, Method 3–Day 2, and Method 3–Day 7, respectively. It was reported that the narrower bandgaps compared to a well-known bandgap of 3.6 eV [2,25] is due to the elevation of the valence-band maximum of SnO_2_ induced by the oxygen vacancies [22,26]. The bandgap of Method 1–Day 8 and Method 2–Day 7 was 3.6 eV.

As has been demonstrated in solar cells, such as perovskite solar cells, sintering SnO_2_ nanoparticles can enhance electron transport properties [14]. Additional studies to evaluate the sintering effects of the obtained SnO_2_ particles on solar cell performance are in progress.

## 4. Conclusions

A low-temperature growth mechanism of SnO_2_ nanoaggregates has been presented for the first time, based on HRTEM, XRD, and XPS analyses of a series of SnO_2_ samples. The changes in the SnCl_2_ and Na_2_CO_3_ concentrations in the reaction mixture enabled the control of the lumpy surface of SnO_2_ at the single-nanometer scale, as well as the Sn (II) formation in the SnO_2_ aggregates. As the sol-gel reaction times increased, the TEM data suggested that the crystallization of tiny nanoparticles was enhanced to form SnO_2_ nanoaggregates with high specific surface areas. In addition, oxygen-vacancy-induced SnO_2_ nanoaggregates could be obtained by adjusting certain factors, including the reaction time and starting material concentrations of SnCl_2_ and Na_2_CO_3_. Such a low-temperature synthesis of 3D SnO_2_ will provide new avenues for developing and producing high-performance functional nanomaterials cost-effectively. Our findings will provide insights for the further investigation of 3D SnO_2_ and its related functions for versatile applications.

## Figures and Tables

**Table 1:** Sizes of SnO_2_ particles in the nanoaggregates observed in the TEM images of Figure 4a–e. The maximum length perpendicular to the lattice fringes of SnO_2_ nanoparticles was measured. The average nanoparticle sizes for the TEM images (**a**)–(**e**) were 2.6 nm, 2.7 nm, 4.4 nm, 3.5 nm, and 4.3 nm, respectively.

Nanoparticles	Size of Nanoparticles (nm)				
	Method 1-Day 5	Method 2-Day 1	Method 2-Day 5	Method 3-Day 1	Method 3-Day 5
1	2.7	2.4	5.0	4.3	4.3
2	2.8	2.6	3.9	3.4	4.2
3	2.0	2.9	4.9	2.6	4.2
4	2.0	2.7	4.3	3.6	4.6
5	3.0		4.0	3.4	4.4
6	3.0		4.4		4.7

**Table 2:** Crystallite sizes and specific surface areas of the SnO_2_ samples synthesized using different reaction conditions and times.

Methods and Days	Crystalline Size (mm)	Specific Surface Area (m^2^/g)
M1D2	2	44
M1D5	3	112
M2D1	3	44
M2D2	3	152
M2D5	5	133
M3D1	3	5
M3D2	3	52
M3D5	3	65
